# Microbiology of the Built Environment: Applying Our Knowledge for Healthy Indoor Spaces

**DOI:** 10.1021/acs.est.6c03510

**Published:** 2026-04-07

**Authors:** Karen C. Dannemiller, Nicholas Nastasi, Anindita Ganguly, Kyle Bibby, Bridget Hegarty

**Affiliations:** † Civil, Environmental, and Geodetic Engineering, The Ohio State University, Columbus, Ohio 43210, United States; ‡ Environmental Health Sciences, The Ohio State University, Columbus, Ohio 43210, United States; § Sustainability Institute, The Ohio State University, Columbus, Ohio 43210, United States; ∥ Environmental Science Graduate Program, The Ohio State University, Columbus, Ohio 43210, United States; ⊥ Department of Civil & Environmental Engineering & Earth Sciences, 6111 University of Notre Dame, Notre Dame, Indiana 46556, United States; # Department of Civil and Environmental Engineering, Case Western Reserve University, Cleveland, Ohio 44106, United States

**Keywords:** bioterrorism, detection, indoor air quality, monitoring

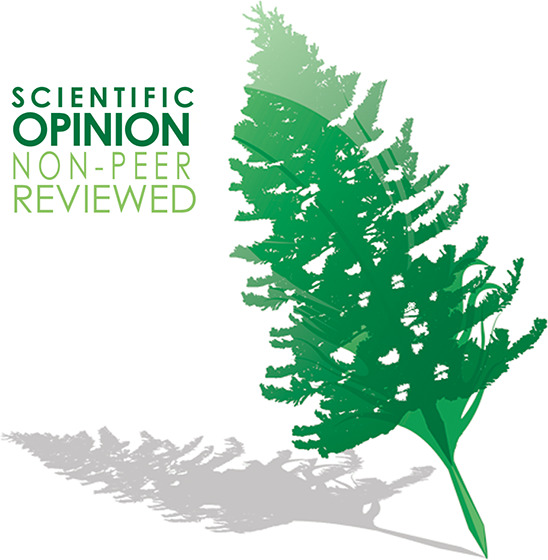

## Introduction

The 2001 anthrax bioterrorism attack in the United States that resulted in 22 cases and 5 deaths^1^ was a critical turning point for our understanding of the organisms that share our indoor spaces. It was immediately clear that new detection technologies were needed to rapidly identify threats. However, we lacked a fundamental understanding of what microorganisms were commonly present in healthy indoor spaces, which made it challenging to identify when there was a problem. Shortly after, the first next-generation sequencing instrument became available and dramatically enhanced our ability to detect the microbial communities in our spaces. The work in the decades that followed resulted in thorough characterization efforts to identify the microbial communities in the built environment under a wide range of conditions.^2^ Events like the COVID-19 pandemic have continued to highlight the importance of understanding indoor microbes. Indeed, we now recognize that microbes in indoor air are not only relevant for the transmission of infectious disease, but also exacerbate chronic conditions such as allergies, asthma, and Chronic Obstructive Pulmonary Disease (COPD).

We are now entering an exciting era in this field where we can implement the information that has been gained to meaningfully improve our indoor spaces to safeguard human health.^3^ Additional studies that simply characterize which microbes are present in certain indoor spaces are likely to provide only a marginal benefit at this point unless testing a specific hypothesis. It is time that we move forward toward implementation of the knowledge we have into usable products and solutions to both limit exposures to harmful microbes and encourage beneficial ones. Below we highlight three active areas for application (Figure 1).

**Figure 1:**
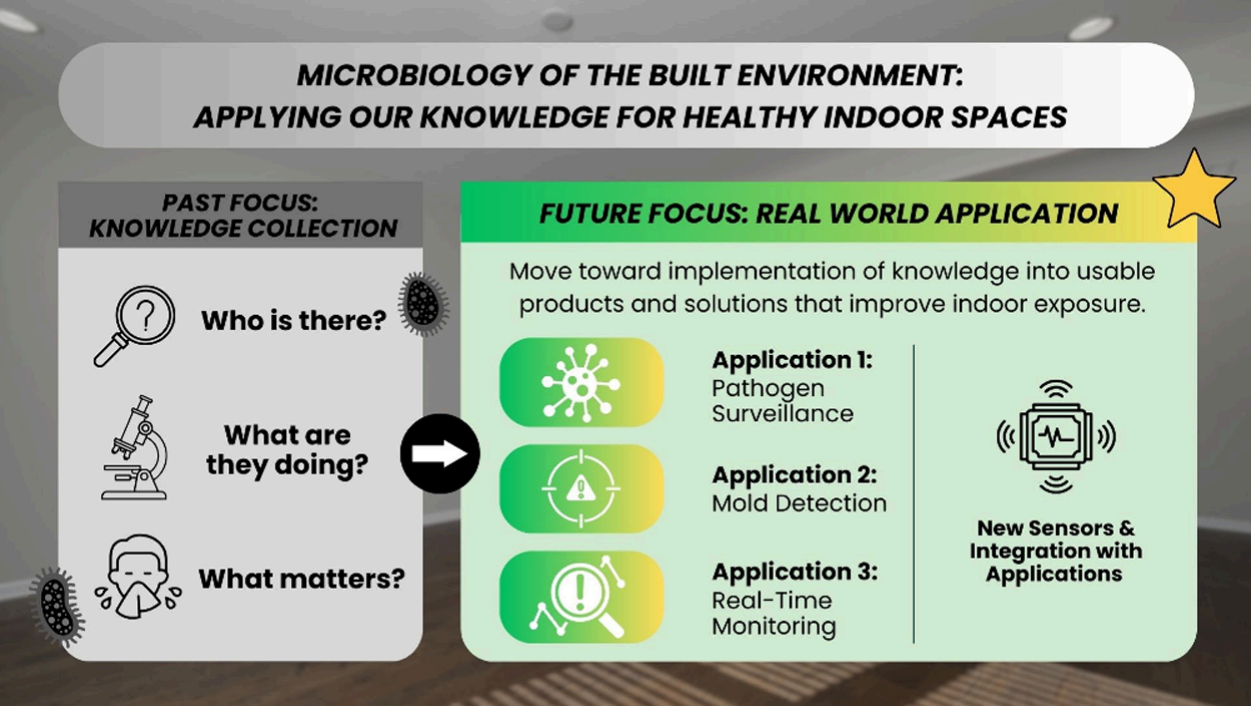
Science of studying the indoor microbiome is undergoing a transition from knowledge collection to real-world application.

## Application 1: Pathogen Surveillance Tools

Transmission of respiratory illness is orders of magnitude higher in indoor spaces compared to outdoor spaces. In addition, we spend the vast majority of our time indoors in developed countries. Thus, shared indoor spaces are critical monitoring locations to minimize disease spread. Responses to the COVID-19 pandemic highlight the potential of environmental monitoring approaches to understand the presence and prevalence of pathogens within communities. While this was most notable with the widespread adoption of wastewater-based epidemiology,^4^ it also motivated the development of pathogen surveillance tools for indoor applications, such as dust^5^ or personal exposure monitors.^6^ A novel recent approach detailed the application of personal facemasks for pathogen monitoring.^7^

We are now better able to put this detection into context of the other microbes that are present and also better understand where pathogens might be located. However, current methods are labor intensive, costly, and generally targeted to a narrow subset of pathogens that may not successfully detect emerging targets. We also need further characterization of the strengths and limitations of different methods to best understand how to collectively use these tools together in different circumstances. Continued technological development is necessary to facilitate widespread adoption to support the vision of real-time indoor air quality monitoring and control.

## Application 2: Enhanced Mold Detection

Neither specific taxa of fungi nor amounts of fungal biomarkers have been consistently connected with negative health effects.^8^ Fungal species vary greatly between buildings even within a small geographic area, which complicates methods to use specific taxa to accurately identify hidden sources of mold. Machine learning based approaches have been able to predict homes with mold damage from amplicon sequencing data, leveraging slight differences in community composition.^9^ And, some commercial kits use amplicon sequencing for identifying homes with mold damage; but, this remains expensive and more work is needed to confirm how broadly applicable these methods are and whether these approaches can be connected with health outcomes.

An alternative approach, suggested by repeated chamber-based experiments, is to focus on fungal metabolism. These metatranscriptomic studies suggest that fungi produce more harmful products at higher humidities, something backed up by pure culture work on aflatoxin and allergen production, potentially explaining the challenge in connecting negative health effects with fungal loads.^10–12^ These differences in gene expression may make it possible to design new, more accurate methods of identifying mold damage in homes based on fungal function rather than species.^10–12^ It is not practical to apply metatranscriptomics as a method of mold detection because it is even more cost prohibitive than amplicon sequencing, but qPCR and other quantification methods would enable the rapid adoption of such technology.

## Application 3: New Sensors and Real-Time Monitoring Strategies

The extensive characterization of microbial communities in the built environment over the past two decades^13^ has helped us understand what is important to monitor. However, sequencing-based approaches are inherently retrospective, and the analysis is often expensive and time-consuming. Now, newer point-of-care and/or real-time monitoring can be designed to provide real-time insights that support healthier indoor environments.

Measuring targets of interest in the built environment remains slow with difficult to understand results. Transitioning to rapid, point-of-care tests using lateral flow immunoassays that target specific targets of interest, combined with a smartphone-based application can provide semiquantitative results within 15 min and can be performed by a homeowner, visiting nurse, or building inspector.^13^ Another promising technology is Loop-mediated isothermal application (LAMP) assays, which is a field deployable molecular technique which can be used to identify targets in typically less than 1 h.

As these rapid and sensor-enabled approaches become more widely adopted, they introduce new bioinformatic and data-integration challenges. Integrating complementary molecular approaches, such as RNA sequencing, may help bridge this gap by providing insight into microbial activity rather than mere presence, enabling more accurate interpretation of real-time signals.

## Conclusions

Now, a quarter century after the 2001 anthrax attacks, we can use the characterizations that have been done to reflect on our current understanding of the distribution of microbes in indoor spaces. The recent advent of novel artificial intelligence and machine learning tools is also creating vast possibilities. In the future, we look toward the implementation of robust tools, monitoring strategies, and interventions that will allow us to improve the microbiology of the built environment to not only control pathogens but better support beneficial microbial communities. Let us see what new technologies the next 5, 10, and 25 years will bring to improve our indoor environmental quality.
